# Surface bound radicals, char yield and particulate size from the burning of tobacco cigarette

**DOI:** 10.1186/s13065-017-0311-3

**Published:** 2017-08-08

**Authors:** Audriy Jebet, Joshua Kibet, Lucy Ombaka, Thomas Kinyanjui

**Affiliations:** 1 Department of Chemistry, Egerton University, P.O Box 536, Egerton, 20115 Kenya; 2 Department of Chemistry, Technical University of Kenya, P.O. Box 52428, Nairobi, 00200 Kenya

**Keywords:** Gas-phase, Free radicals, Particulate matter, Reactive intermediates, Tobacco

## Abstract

**Background:**

Tobacco smoke is a toxic gas-phase cocktail consisting of a broad range of organics, and free radical intermediates. The formation of smoke from a burning cigarette depends on a series of mechanisms, including generation of products by pyrolysis and combustion, aerosol formation, and physical mass transfer processes.

**Methods:**

The current study simulates the deposition of particulate matter on the human lung surface by trapping the tobacco smoke particulates in situ on silica gel. To mimic this phenomenon, the cigarette was smoked directly on siliga gel. The surface morphology of smoke condensate trapped on silica gel, and pure silica gel (control) was investigated using a scanning electron microscope (SEM). Electron paramagnetic resonance (EPR) was used to explore the presence of free radicals on the particulate matter trapped on silica. Standard procedures for cigarette smoking (ISO 3402:1999) were adopted. The char yields of tobacco cigarette in the temperature range 200–700 °C was also investigated in an inert atmosphere using a quartz reactor.

**Results:**

SEM images showed the surface morphology of pure silica gel was smooth while silica gel on which cigarette smoke was smoked on contained particulates of various sizes. Generally, the particulate size of cigarette smoke adsorbed on silica was found to be 2.47 ± 0.0043 µm (~PM_2.5_). Electron paramagnetic resonance (EPR) results showed a g-value of 2.0037 typically that of a carbon-centred radical.

**Conclusions:**

It is therefore evident from this investigation that cigarette smoke contains surface bound radicals considered harmful to the health of cigarette smokers. The particulate size of tobacco smoke (PM_2.5_) can impact severely on the lives of the cigarette smoking community because of its near ultrafine nature. This significantly small particulate size in cigarette smoke can be inhaled deeper into the lungs thus causing serious cell injury and possible tumour growth in addition to other grave diseases.

**Graphical abstract:**
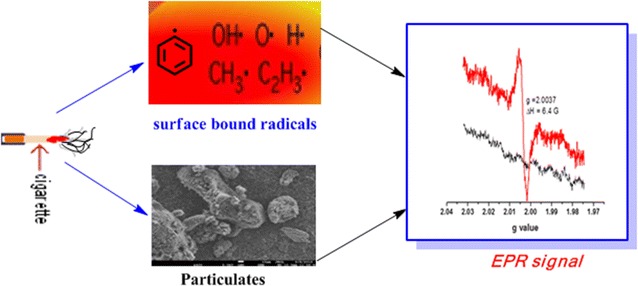
Cigarette smoking and analytical techniques employed in this study

## Background

The radical immobilization of reactive intermediates on a surface is a remarkable area of study and has recently attracted enormous interest not only in tobacco research but also in combustion science. This work is important since it provides evidence on radical trapping on a silica surface exposed in situ by cigarette smoke through deposition of particulate matter. The radicals identified as carbon-centred ones may, indeed, easily end-up on human lung surface being transported by various sizes of particulate matter (PM) [1–3]. Moreover, tobacco use in form of cigarettes has gained unprecedented popularity worldwide despite massive evidence that it is one of the primary causes of death among the cigarette smoking community. It is well established in literature that numerous physical processes and chemical reactions occur inside the burning zone of a cigarette resulting to release of organic toxins, intermediate free radicals and other bio-hazardous by-products [4, 5].

Cigarette smoke is an aerosol of liquid droplets (the particulate phase) suspended within a mixture of gases and semi-volatile compounds believed to contain surface bound radicals responsible for serious lung damage, and are considered precursors for a variety of ailments including cancer and cardiopulmonary death [1]. Some of the intermediate radicals such as benzyl, phenoxy and semiquinone type radicals produced from tobacco burning are resonance stabilized and are environmentally persistent free radicals (EPFRs) exhibiting long lifetimes and consequently potential candidates for extensive cellular aberrations in cigarette smokers [6, 4]. We are aware that many studies on tobacco radicals have been studied previously but not in the manner this investigation has done. Whereas previous studies by Church and Pryor [2] explored tobacco free radicals using spin trapping techniques and radical extracts from Cambridge filter pad using various solvents, this study reports surface immobilization of cigarette smoke radicals in situ on silica gel. Nevertheless, this is not to say our study is in contradiction of the work done by Church and Pryor [2], and Maskos et al. [7] but rather underscore the existence of tobacco free radicals in tobacco particulates in a way that could mimic actual cigarette smoking.

Two kinds of smoke with different composition and properties have been classified in literature during cigarette smoking: mainstream smoke inhaled by the smoker and side stream smoke, which is released into the environment between puffs from the burning end of the cigarette [3]. Side stream smoke (generally known as environmental tobacco smoke, ETS) escapes into the surrounding air a long with the gas-phase, diffusing into the cigarette paper and makes side-stream emissions ([8, 9]). Formation of smoke from a burning cigarette depends on a series of mechanisms, including generation of products by pyrolysis and combustion, aerosol formation, and physical mass transfer processes [10]. Tobacco smoke is a very complex cocktail consisting of over 6000 compounds representative of a chemical reactor where several intricate chemical processes take place during pyrolysis [11–15]. The mainstream smoke emitted from the mouth end of a burning cigarette is mainly produced by combustion and pyrolysis reactions as well as distillation processes in the burning tip of the cigarette when the cigarette is puffed out [16].

Previously, studies have indicated that approximately 10^6^ alkyl- and alkoxy radicals could be present in the gas-phase of one cigarette, or 5 × 10^14^ radicals per cigarette puff [17]. In this study the radical characteristics especially the carbon cantered radicals, tobacco char and the surface morphology of cigarette smoke particulate is explored. EPR spectral identification parameter apparent g-value (maximum point of the integrated curve) was used to identify the radical in tobacco smoke particulates. The biological and environmental consequences of free radicals occasioned by the formation of reactive oxygen species (ROS) on tobacco particulates is a grave concern to the cigarette smoking community because the labile species being produced during physico-chemical processes of tobacco burning as free radical intermediates may initiate serious health impacts in the biological environment since their ability to generate of ROS may cause oxidative stress in living organisms. Production of ROS can results in severe oxidative stress within cells via the formation of oxidized cellular biological molecules such as lipids, proteins, and DNA [18]. These radicals are capable of causing biological damage in human cells [1, 4]. As a result, free radicals from cigarette smoke can be taken as the major precursors for the generation of ROS considered injurious to human health.

From a toxicological standpoint, the particulate and the gas-phase of tobacco smoke contains many poisonous, carcinogenic and mutagenic chemicals, as well as stable and unstable free radicals with the potential for biological oxidative damage to human organisms hence this study is necessary [5]. This study therefore presents unique data on the particulate characteristics of tobacco char, the persistent free radical on tobacco smoke particulate, and the particulate size distribution of tobacco smoke. Although there are many complex parameters at play during cigarette smoking such as the gas-phase/liquid interface, we believe this study may simulate the characteristic behaviour of the average particulate size deposited in the lung tissues of cigarette smokers. Additionally, this contribution is part our sister articles on tobacco research aimed at providing an important piece of knowledge towards understanding tobacco [15, 5]. Ultimately, tobacco smoke particulates and radicals are well established xenobiotics and eco-toxicants.

## Methods and materials

The heater (muffle furnace) was purchased from Thermo Scientific Inc., USA while the commercial cigarettes (for confidential reasons named SM1 and ES1) were purchased from a retail outlet and conditioned at room temperature under constant humidity in accordance with tobacco smoking protocol established by Fresenius [19] and more recently by Bush et al. [20]. The average length of the filter for the cigarette under study was 2.2 cm. This is the standard length of filters for most commercial cigarettes sold in Kenya as regulated by the Kenya Bureau of Standards (KEBs). SM1 cigarette smoke adsorbed on silica was investigated for surface bound radicals. Methanol, dichloromethane (DCM), and silica gel (size 150–200 µm) were of analytical grade (≥99% purity) and purchased from Sigma Aldrich Inc. (St. Louis, Missouri, USA).

### Adsorption of cigarette smoke on silica surface

**Fig. 1:**
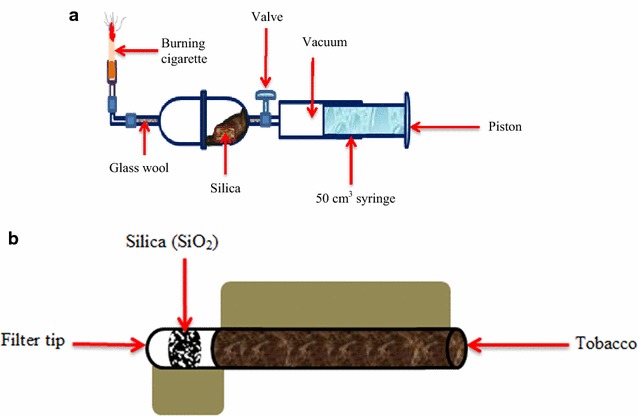
**a** Experimental set up for particulate trapping on silica gel. **b** Illustration showing how silica was sandwiched between the filter and tobacco

Five (5) conditioned cigarette sticks were smoked directly onto silica gel as presented in Fig. 1a. To ensure consistency during cigarette smoking, five experiments were conducted and the yield of tobacco particulate was calculated in each case. Silica of mass between 30 and 31.6 mg was weighed and placed in the smoking apparatus (cf. Fig. 1a). The % weight of total particulate matter per cigarette after smoking was then calculated and presented in Table 1, vide infra. On the other hand, the amount of particulate matter adsorbed on the silica gel sandwiched between the cigarette filter and tobacco was investigated for comparison. 30 mg of silica was sandwiched between the filter and tobacco as presented in Fig. 1b. In this case, five cigarette sticks of cigarette SM1 and other five cigarettes sticks were smoked for cigarette ES1. The yields of particulate matter for each cigarette SM1 and ES1 were determined and compared as shown in Table 2.

**Table 1:** The yields of total particulate matter from SM1 cigarette smoked directly on silica gel

Experiment	Mass of silica gel before smoking (mg)	Mass of silica gel after smoking (mg)	Particulate matter (mg)	% yield of particulate matter	% yield per cigarette
1	30.0	33.4	3.4	11.33	2.27
2	30.5	34.5	4.0	13.11	2.62
3	31.0	34.8	3.8	12.26	2.45
4	30.4	33.9	3.5	11.51	2.30
5	31.6	36.0	4.4	13.92	2.78
Mean	30.70 ± 0.62	34.52 ± 0.99	3.82 ± 0.40	12.43 ± 1.09	2.49 ± 0.22

**Table 2:** The yields of total particulate matter from SM1 and ES1 cigarettes smoke when silica gel was sandwiched between the filter and tobacco

Experiment	Mass of silica gel before smoking (mg)	Particulate matter of ES1 (mg)	Particulate matter (mg) of SM1	% yield per cigarette of ES1	% yield per cigarette of SM1
1	30.0	2.3	2.1	12.4	11.9
2	30.0	2.9	2.9	14.3	14.3
3	30.0	2.9	2.6	14.3	13.3
4	30.0	3.6	1.9	16.7	11.0
5	30.0	2.4	3.1	12.9	15.2
Mean	30.0	2.80 ± 0.45	2.5 ± 0.50	14.5 ± 1.50	13.1 ± 1.55

All experiments were conducted in a fume chamber to reduce the health problems associated with cigarette smoking. A cigarette stick was stuck at the tip end of rubber tubing and lit using a cigarette lighter (Fig. 1a). To ensure constant burning of the cigarette under ambient conditions, a 50 mL syringe was used to draw in air to the burning cigarette. The cigarettes were smoked at a rate of 35 mL/2 s once every 60 s according to ISO 3402:1999 standards [20], based on a series of investigations on the smoking characteristics of cigarette consumers by Fresenius [19]. An apparent poisonous matrix of tobacco particulate described in detail in our earlier works and previous studies [6, 5, 7] was adsorbed on the surface of silica gel and analysed using EPR and SEM spectroscopic techniques.

### Experimental protocol for electron paramagnetic resonance spectroscopy

Smoke particulate from the burning of tobacco cigarette (SM1) trapped in situ on silica and immediately analyzed using EPR. About 5 mg sample of SM1 tobacco particulate adsorbed on silica gel was analyzed using a Bruker EMX-20/2.7 EPR spectrometer (X-band) with dual cavities, modulation and microwave frequencies of 100 kHz and 9.516 GHz, respectively [21, 4]. The characteristic parameters were: sweep width of 200 G, EPR microwave power of 1–20 mW, and modulation amplitude of ≤4 G. The time constant was varied appropriately. The sweep time was set at 84 s and the number of scans was fixed at 10. The receiver gain for this investigation was 50. The g-value was computed using Bruker’s WINEPR program, which is a comprehensive line of software, allowing control of the Bruker EPR spectrometer, data acquisition, automation routines, tuning, and calibration programs on a Windows-based PC [22, 4]. The exact g-value for the key spectrum was determined by comparing with a 2,2-diphenyl-1-picrylhydrazyl (DPPH) standard [23]. Constituent radicals in a mixture can only be distinguished by judicious variation of the experimental conditions (temperature, pressure, and annealing parameter procedures) followed by computer analysis of digitally stored spectra [21] which is beyond the scope of the current investigation.

### The thermal degradation of tobacco

In order to investigate the mass loss characteristics of tobacco during cigarette smoking, 30 mg of tobacco was pyrolyzed in a flow of N_2_ in a quartz reactor of dimensions: i.d. 1 cm × 2 cm housed in an electrical heater under conditions that simulate cigarette smoking [20]. The pyrolysis temperature was varied at intervals of 100 °C between of 200–700 °C at a constant residence time of 2.0 s for a total pyrolysis time of 3 min. The residue formed at every pyrolysis temperature was collected and weighed in order to determine the yields of char. The % yield of char was then plotted as a function of pyrolysis temperature.

### Scanning electron microscopy analysis

The silica gel containing adsorbed tobacco particulate on its surface was dissolved in dichloromethane through a porous tube diluter and transferred into amber vials for SEM analysis. About 5 mg of the particulate sample was added to 1 mL methanol and gold grids were dipped into the prepared sample. Twisters were used to pick the gold grids from the sample [24]. The grid containing the sample was allowed to dry in the open air before inserting it into the analysis chamber of the SEM (JEOL JMS 7100F) operated at 5 kV [25]. The sample was analysed under high vacuum to ensure no interference of air molecules during analysis. The SEM machine was then switched on and imaging of the sample conducted at 5.0 kV using a light emitting diode (LED). The lens was varied at various resolutions until a clear focus of the sample was observed. The size of particulates was measured using *image J* program and the data was plotted using *Igor* graphing software [24]. Three micrographs from three experiments were used in SEM analysis in order to have reasonable points for statistical averaging. The average size of particulates was generated directly by *Igor* graphing software. Igor is state-of-the art software in drawing graphs and has special in-built codes for computing averages, standard deviations, and curve fitting features. The particulate size of cigarette smoke in this study is taken as the mean diameter of all the particulates measured using *image J*.

## Results and discussion

### Decomposition profile of tobacco

**Fig. 2:**
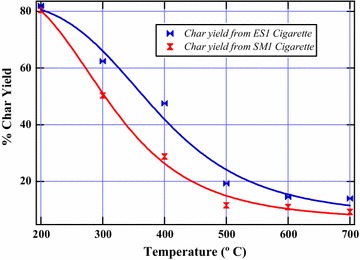
Wt% yield of char from the thermal degradation of tobacco from SM1 cigarette (*red curve*) and ES1 cigarette (*blue curve*)

The thermal decomposition behaviour of cigarette tobacco in an inert environment (under N_2_) over the temperature range of 200–700 °C produced interesting results as shown in the Fig. 2. The char yield follows a decay pattern as temperature increases. The initial sharp decrease in % char to ~80% for both cigarettes (SM1 and ES1) at 200 °C may be attributed to high mass loss of water and other volatiles such as CO_2_, CO and possibly methane in the tobacco sample [4]. Significant mass loss was also registered between 200 and 400 °C for SM1 was ~50% while that of ES1 in the same temperature range was ~32%. This is consistent with other results on tobacco pyrolysis which indicate high mass loss of tobacco pyrolysis occurs in this temperature region [26].

The mass loss between 400 and 500 °C was ~17% for SM1 tobacco cigarette whereas that of ES1 tobacco cigarette was ~28%. This region coincides with the highest release of molecular toxins as reported in literature [4, 26]. At 500 °C most molecular organics will have been evolved so that any further increase in temperature leads to a sharp decrease in the pyrolysis by-products. These results are remarkably consistent with previous data reported in literature on biomass pyrolysis [6, 4]. Any further increase in temperature (above 500 °C) did not yield significant change in mass. The lowest mass loss was recorded at 700 °C (~10% for SM1 and 13% for ES1 cigarettes respectively). Predictably, char yield at high temperatures was observed to remain constant because the residue at these temperatures is largely carbonaceous and nearly all molecular by-products are understood to have been evolved. The difference in the decomposition profiles for the two cigarettes investigated could be mainly due to the ingredients added during cigarette manufacture and the different growing conditions of tobacco used in cigarette processing [5]. We have articulated previously that in order to minimize organic toxins evolved during cigarette smoking there is need to design cigarettes that can be smoked at lower temperatures of about 300 °C or less [6, 5].

### Electron paramagnetic resonance results

**Fig. 3:**
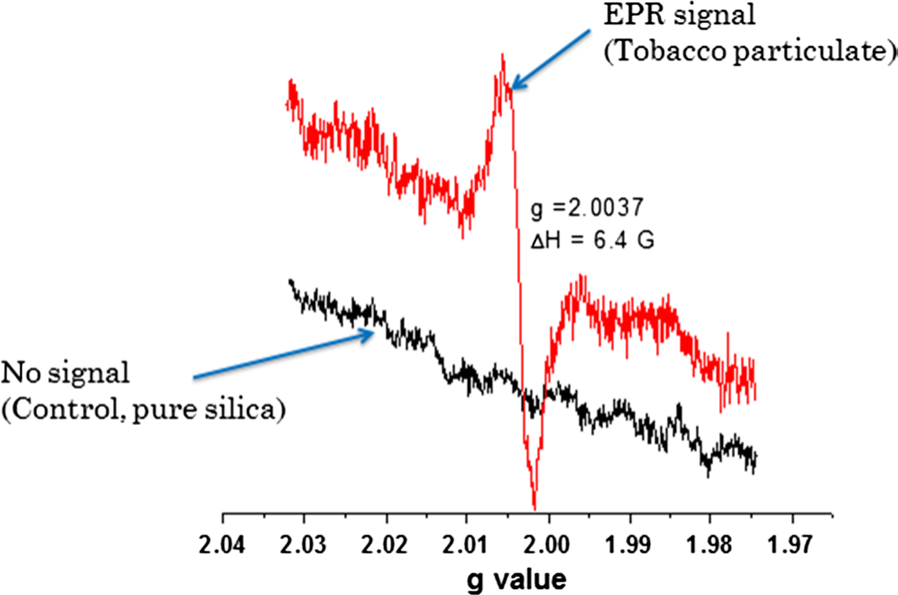
Particulate matter signal from cigarette smoke adsorbed on silica gel (*red line*) and signal from pure silica gel (control), *black line*

The signal displayed a g-value of 2.0037, typical of carbon centred radicals such as benzyl, biphenyl ether or phenoxy type radicals, or a semiquinone-type radical possibly from aromatic type compounds in a complex matrix such as plant matter [27, 28]. Figure 3 shows the EPR signal for the particulate matter adsorbed on silica and the control (no signal—pristine silica). However, the g-value does not necessarily give conclusive structural information when the EPR spectrum is a convolution of two or more species [21]. Therefore the signal presented in Fig. 3 may comprise superimposed radicals of various organics despite the carbon cantered radical being significantly dominant. This is because tobacco plant consists of various components such as lignin, cellulose, amino acids, and pectin [6]. The ΔH_P-P_ for the radical detected in the particulate matter of tobacco was narrow (ΔH_P-P_ = 6.4 G). The spectrum was an unstructured with some anisotropy [4]. Nevertheless, the EPR signal may be a superimposition of various radicals considering the fact that the pyrolysis of tobacco plant may consist of various intermediate radicals ranging from phenoxy type radicals, benzyl centered radicals, to quinone type radicals or the occurrence of strong matrix interactions between pyrolysis by-products of tobacco biomass [21, 7].

The control (pure silica gel) showed no EPR signal. This validates our proposition that radicals are indeed present in tobacco particulate as surface bound reactive intermediates. Radicals in tobacco smoke are considered responsible for initiating the production of reactive oxygen species (ROS) widely believed to cause severe illnesses among cigarette smokers. For instance the hydroxyl radicals are powerful oxidizing agents responsible for mutation and damage of essential macromolecules in the biological environment [29]. Nevertheless, the tobacco industry is progressively subjected to national and international guidelines with consumer safety in mind [3] but this is yet to bear dividends despite the intensity directed towards tobacco research since the 1950s [19].

### Percentage yield of tobacco smoke particulates adsorbed on silica gel

The % yield of total particulate matter (TPM) trapped on silica per cigarette after cigarette smoking (Fig. 1a) was calculated and found to have a mean of ~2.49% per cigarette (Table 1). It is remarkable that only a small fraction of cigarette particulate was adsorbed on silica. This may be attributed to the few adsorption sites on silica or the complex behaviour of tobacco which is yet to be understood. During smoking, nonetheless; not all smoke puffed in by the smoker is adsorbed in the lungs because possibly most of it is puffed out. Clearly, this observation has revealed that most of the particulate matter (~98% per cigarette) may be puffed out by the cigarette smoker and only about 2% per cigarette probably get absorbed on the lung surface of the smoker according to this study. However, the lung surface may have a high number of adsorption sites than the silica gel used in this work and thus accurate simulation of smoke deposition in the lung surface may not be possible. Nevertheless, this work provides a basis for further investigation on the practicability of experiments and machines designed to simulate actual cigarette smoking.

On the other hand, when a small piece of the filter was carefully cut and replaced with silica (Fig. 1b), about 13% of the total particulate matter per cigarette for SM1 cigarette was retained by silica, leaving ~87% to pass through the filter while about 15% of total particulate matter per cigarette for ES1 cigarette was retained leaving approximately 85% TPM to pass through the filter (Table 2). Evidently, an adsorbent incorporated into the filter should reduce the amount of total particulate matter impinging on the lungs of the cigarette smoker. This information is therefore critical in designing filters which have the potential to adsorb cigarette particulate matter and therefore reduce the amount of smoke particulates getting into the lungs of a cigarette smoker.

### Surface morphology of tobacco smoke particulates

**Fig. 4:**
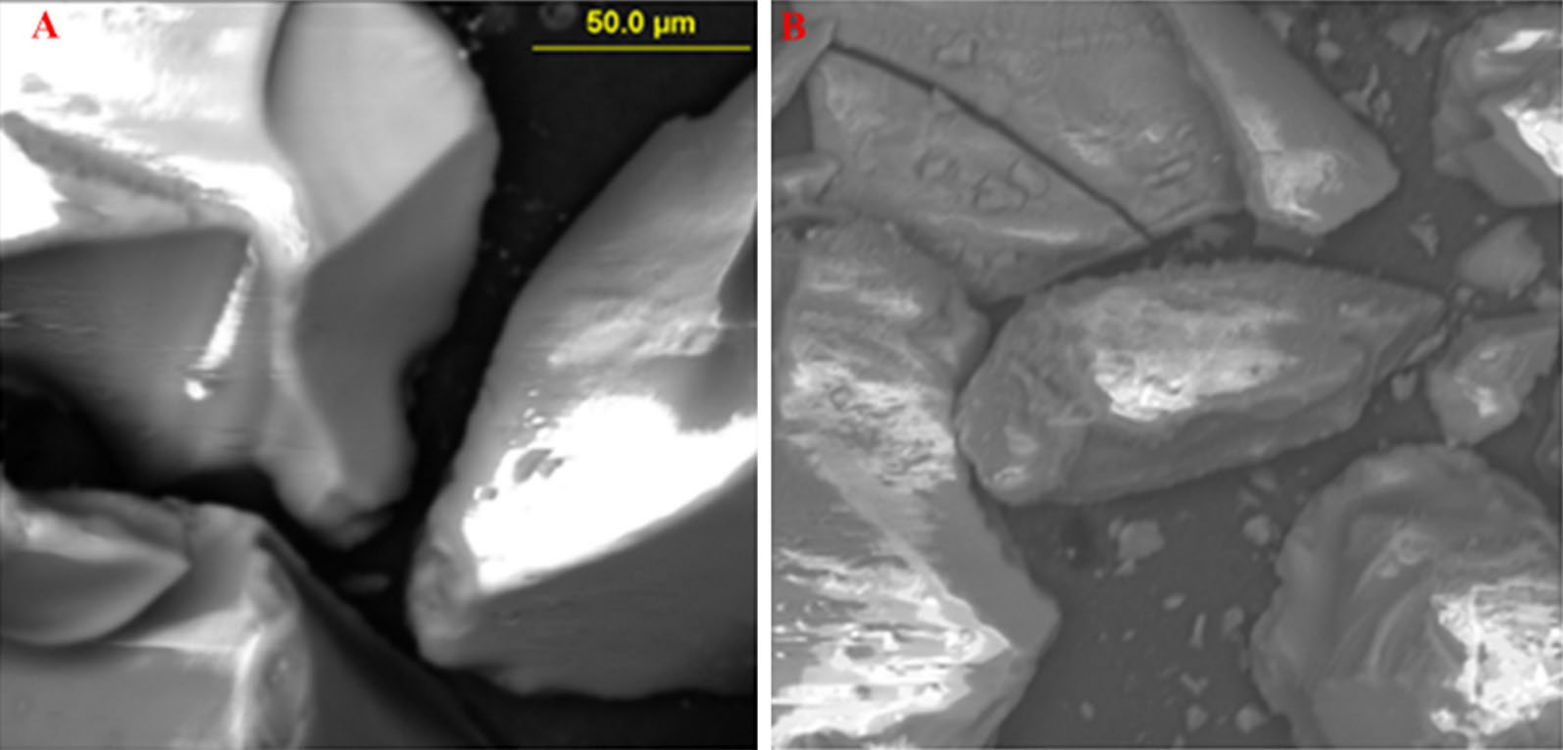
SEM image of pristine silica (SiO_2_) before adsorption (**A**) and SiO_2_ after cigarette smoke adsorption (**B**)

**Fig. 5:**
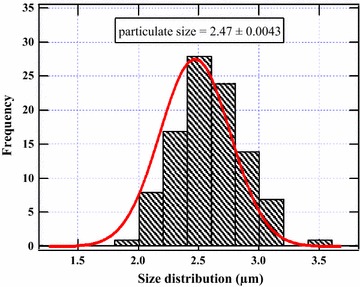
Size distribution of cigarette smoke particulates adsorbed on silica gel

SEM image of the pure silica gel and the cigarette smoke adsorbed on silica gel is presented in Fig. 4 at an attendant magnification of 400× at 50 µm. The images displayed the morphology of silica gel as smooth and glassy surface with some mineral impurities. The particle size of cigarette smoke as reported in this study was ~2.47 ± 0.0043 µm (Fig. 5). Therefore, the particulate size from cigarette smoke falls well under PM_2.5_ classification of particulate matter as predicted in this work. Particles of this size are known to cause severe respiratory illnesses including lung cancer, bronchitis, and cardiac arrest because of their approximate ultrafine nature [30]. Particulate matter from tobacco smoke and other combustion sources generally consists of small particles distributed in the air, and may have enormous harmful effects on human health and natural ecosystems as a whole. It is well established in literature that PM can cause premature death, cardiovascular damage, decreased lung function, and respiratory infections and cancer related illnesses [31, 32]. Thus the smaller the particulate matter as is the case in this study, the more severe the health effects are likely to be on the cigarette smokers.

Particulate matter in general is largely a mixture of solid particles and/or liquid droplets that may be approximately 2.5 μ or less. The results presented in this work corroborate previous studies reported in literature on the size of particulate matter of tobacco smoke [33]. On the other hand, PM_10_ which may also be present in tobacco smoke due to conglomeration of smoke units comprise particles of between 2.5 and 10 µm, which are inhalable as coarse particles [33]. Nonetheless; particulate size of tobacco smoke has also been reported in literature to be ≤1 µm [19] for fresh tobacco. Consequently, the difference between our results and earlier data on particulate size of cigarette smoke may be attributed to the type of tobacco, tobacco aging processes, and the adsorbent used. In our study, the tobacco explored may be considered “aged” implying that the cigarette sample might have been in the retail shelf for unspecified period of time prior to this investigation. Remarkably, cigarette conditioning, aging, and cigarette type (depending on the ingredients added to the tobacco during processing, and the tobacco growing conditions) may have a profound effect the size of the particulates emitted during cigarette burning. Inhalation and deposition of cigarette smoke particulates in the respiratory system may result in the release of reactive oxygen species (ROS) implicated in a series of ailments affecting tobacco consumers particularly due to surface bound radicals and other reactive intermediates present in tobacco smoke.

## Conclusions

The current study has demonstrated that tobacco cigarette particulate matter contains surface bound free radicals which can be detected in situ during cigarette smoking. The free radicals identified in this investigation could comprise carbon-centered benzyl type radicals—dominant in this work, and possibly oxygen-centered (semiquinone or phenoxy type radicals) aromatic species because of their potential delocalization within a π system. This work also attempted to simulate how tobacco smoke particulates (containing intermediate free radicals) may be deposited on the lung tissues during cigarette smoking. Furthermore, it was found that an average of approximately 2.5 µm tobacco particulates was deposited on silica gel during cigarette smoking after passing through the cigarette filter. The particulates were generally within the classification of PM_2.5_. Besides, the thermal degradation of tobacco presented in this study showed that the greatest mass loss occurred between 300 and 400 °C which coincides with the temperature at which significant organic volatiles are released as documented in our previous studies and in literature thus designing cigarettes that can be smoked at lower temperatures (≤300 °C) would be beneficial to the cigarette smoking community. We submit that because of the complex nature of the airway system (air/liquid interface and adsorption sites on the lung surface) it may not be possible to design smoking conditions that simulate accurately the behaviour of cigarette smoke particulates that impinge on the surface of the lungs during cigarette smoking. Nonetheless, the results presented in this work are remarkably interesting and advance critical information on the current body of knowledge on tobacco research.
